# Erratum: verification of the accuracy of a hybrid breast imaging simulation framework for virtual clinical trial applications

**DOI:** 10.1117/1.JMI.7.6.069801

**Published:** 2020-12-29

**Authors:** Liesbeth Vancoillie, Nicholas Marshall, Lesley Cockmartin, Janne Vignero, Guozhi Zhang, Hilde Bosmans

**Affiliations:** a KU Leuven, Division of Medical Physics and Quality Assessment, Department of Imaging and Pathology, Leuven, Belgium; b UZ Leuven, Department of Radiology, Leuven, Belgium

## Abstract

The erratum adds funding information missing from the Acknowledgments section of the originally published version of the article.

This article [*J. Med. Imag.* **7**(4), 042804 (2020) doi: 10.1117/1.JMI.7.4.042804] was originally published in Vol. 7, Issue 4 of the *Journal of Medical Imaging* (JMI) on 22 April 2020 with information missing from the Acknowledgments section. Specifically, the authors mistakenly omitted acknowledgment of financial support from FFG-FWO, Grant No. 861552. The article was republished with the correct, complete funding information on 21 December 2020.

